# Removal of Methylene Blue and Congo Red Using Adsorptive Membrane Impregnated with Dried *Ulva fasciata* and *Sargassum dentifolium*

**DOI:** 10.3390/plants10020384

**Published:** 2021-02-17

**Authors:** Ahmed Labena, Ahmed E. Abdelhamid, Abeer S. Amin, Shimaa Husien, Liqaa Hamid, Gehan Safwat, Ayman Diab, Adil A. Gobouri, Ehab Azab

**Affiliations:** 1 Egyptian Petroleum Research Institute (EPRI), Nasr City, Cairo 11727, Egypt; shimahessin@yahoo.com; 2 National Research Centre (NRC), Dokki, Cairo 12622, Egypt; ae.abdel-hamid@nrc.sci.eg; 3 Botany Department, Faculty of Science, Suez Canal University, Ismailia 41522, Egypt; abeeramin2003@yahoo.com; 4 Faculty of Biotechnology, October University for Modern Sciences & Arts (MSA University), 6th October City 12566, Egypt; leqaa.essam@msa.edu.eg (L.H.); gsafwat@msa.eun.eg (G.S.); aymanalidiab@gmail.com (A.D.); 5 Department of Chemistry, College of Science, Taif University, P.O. Box 11099, Taif 21944, Saudi Arabia; a.gobouri@tu.edu.sa; 6 Department of Nutrition and Food Science, College of Science, Taif University, P.O. Box 11099, Taif 21944, Saudi Arabia; e.azab@tu.edu.sa; 7 Botany and Microbiology Department, Faculty of Science, Zagazig University, Zagazig 44519, Egypt

**Keywords:** biosorption, adsorptive membrane, dye removal, *Ulva fasciata*, Sargassum dentifolium, textile effluents, Methylene blue, Congo red

## Abstract

Biosorption is a bioremediation approach for the removal of harmful dyes from industrial effluents using biological materials. This study investigated Methylene blue (M. blue) and Congo red (C. red) biosorption from model aqueous solutions by two marine macro-algae, *Ulva fasciata* and *Sargassum dentifolium,* incorporated within acrylic fiber waste to form composite membranes, Acrylic fiber-*U. fasciata* (AF-U) and Acrylic fiber-*S. dentifolium* (AF-S), respectively. The adsorption process was designed to more easily achieve the 3R process, i.e., removal, recovery, and reuse. The process of optimization was implemented through one factor at a time (OFAT) experiments, followed by a factorial design experiment to achieve the highest dye removal efficiency. Furthermore, isotherm and kinetics studies were undertaken to determine the reaction nature. FT-IR and SEM analyses were performed to investigate the properties of the membrane. The AF-U membrane showed a significant dye removal efficiency, of 88.9% for 100 ppm M. blue conc. and 79.6% for 50 ppm C. red conc. after 240 min sorption time. AF-S recorded a sorption capacity of 82.1% for 100 ppm M. blue conc. after 30 min sorption time and 85% for 100 ppm C. red conc. after 240 min contact time. The membranes were successfully applied in the 3Rs process, in which it was found that the membranes could be used for five cycles of the removal process with stable efficiency.

## 1. Introduction

Water pollution is considered a significant global problem, recently causing destruction of the ecosystem and having significant effects on human health. Water pollution due to industrial effluents is the most significant type of pollution because of rapid recent industrialization and technological development. Textile industries are an example of industry that uses a large amount of dye, such as Methylene blue (M. blue) and Congo red (C. red) [1,2]. These producers discharge their effluents into water streams, resulting in an adverse effect on the environment, i.e., water living organisms, plants, and animals, in addition to humans [3,4]. Many physico-chemical technologies, such as adsorption, chemical precipitation, ion exchange, and membrane filtration, have been proposed for the removal of dyes from polluted wastewater [5,6,7,8]. However, these technologies are ineffective at small scales due to their high cost and the large amount of hazardous waste that may be generated by their used [9]. Therefore, interesting solutions have been applied using biological masses, such as bacteria, fungi, agriculture wastes, and algae, for pollutant removal in a process known as a biosorption [10,11,12,13,14,15]. Algal biomasses, as a type of biological biomass (dried form), have attracted significant interest of researchers due to their availability and ease of handling. Many types of algae, such as *Chlorella* sp., *Nostoc* sp., *Sargassum dentifolium*, *Scendesmus* sp., and *Anabeana* sp., have been investigated [16,17,18,19,20]. Various algal biomasses have been investigated in the removal process of different forms of dyes, such as Crystal violet, Malachite green, Methylene blue, and Congo red. However, although a variety of algal species have been found to be potent sorbent materials, their use in the removal process has been lacking, due to difficulties in their harvesting techniques arising from their powder form. Therefore, the search for new alternative approaches, such as algal incorporation with polymeric substances, aims to simultaneously provide an easy harvesting process and better removal efficiency. Many polymeric materials, both synthetic or natural, can be used in different forms, such as beads, powders, filaments, and membranes with a controlled morphology. Acrylic fibers (AF) are one of the most used polymers in many textile products, such as cloths and carpets [21,22]. Acrylic fibers comprise at least 85% acrylonitrile, with the remainder comprising another acrylic monomer, mainly acrylic ester, added during the manufacture of the fiber to improve certain characteristics, such as dyeability or processing. Fiber waste may cause environmental and economic problems. Several research papers reported the recycling of acrylic fiber waste via chemical treatment of nitrile groups or grafting techniques [21,23]. These techniques involve high cost and significant energy use and produce byproducts that cause environmental problems.

In this study, we used polymeric waste in combination with algal biomass to remove different dyes from aqueous solution. Acrylic fiber wastes were used with *Ulva fasciata* and *Sargassum dentifolium* algal species, in dried form, to form two composite membranes for Methylene blue (M. blue) and Congo red (C. red) removal from synthetic aqueous solutions. Three types of membranes were obtained: Acrylic fiber (AF) as a blank, acrylic fiber incorporated with *Ulva fasciata* (AF-U), and acrylic fiber incorporated with *Sargassum dentifolium* (AF-S). The optimization process was displayed on the membrane to achieve the highest M. blue and C. red removal efficiency. Characterization analyses were applied to the membranes, namely, FT-IR and SEM. Additionally, kinetics, isotherm, and desorption studies were undertaken. Another aim of this study was the investigation of the static mode on the adsorption/biosorption process, in which all experiments were performed in a static manner. This model was applied to help achieve high pollutant removal with a low cost-process, achieved via minimization of the cost of the adsorption steps. Additionally, the 3Rs process (i.e., removal, recovery, and re-use) was investigated.

## 2. Results and Discussion

### 2.1. One Factor at a Time (OFAT) Method (First Optimization Step)

#### 2.1.1. Effect of Contact Time

Different contact times of 0.5, 1, 2, 3, 4, 8, and 12 h were investigated for M. blue and C. red removal using AF-U and AF-S membranes in a static model with 100 ppm dye conc. and pH 7. Figure S1a illustrates M. blue and the C. red removal by the AF-U membrane, in which maximum removal rates of nearly 92% and 80% were achieved for M. blue and C. red, respectively. Removal efficiency of nearly 85% and 80% was recorded for M. blue and C. red, respectively, using the AF-S membrane, as shown in Figure S1b. Furthermore, it was noted that the M. blue removal efficiency increased by increasing the contact time to 2 h for the AF-U and 4 h for the AF-S membrane; subsequently, the removal rose slightly. For C. red, the removal took a longer time for both membranes, with efficiency achieved after 4 h. In addition, the M. blue removal efficiency was higher than the C. red removal efficiency in both types of membranes [24,25,26]. This result can be attributed to the negative nature of the membranes’ functional groups that are represented in the proteins, carotenoids, polysaccharides, lipids, and phenolic compounds of the membranes [27,28]. These functional groups are carboxyl, amino, sulfonyl, hydroxyl, and carbonyl groups that are important for the dye-binding process, particularly of the cationic dyes (M. blue) [28].

#### 2.1.2. Effect of pH

Different pH values were investigated for both types of membranes, AF-U and AF-S, for M. blue and C. red removal. It was noted that there is no recognizable difference between the removal values at different pH values. However, the highest M. blue and C. red removal efficiencies were achieved at pH 5. This pH value was fixed for all of the subsequent experiments. Additionally, it was observed that C. red was turned into a violet color, and then a blue color, and subsequently precipitated under an acidic condition (around pH 3). No result was recorded at this pH for C. red removal (see Figure S2a,b). This observation may be attributed to the hydrophobic interaction between the dye molecules’ aromatic rings that caused a π–π stacking phenomenon, which resulted in agglomeration and precipitation of this dye under the acidic condition [29,30].

#### 2.1.3. Effect of Algal Dose in the Membrane

The dose of algae is the most important parameter that affects the removal capacity. In the current study, different percentages of *U. fasciata* and *S. dentifolium* in the AF membrane (0, 5, 10, and 20%) were investigated for M. blue and C. red removal. Figure S3a,b shows that 20% is the optimum algal percentage that achieved the highest M. blue and C. red removal efficiencies. This result may be related to the increase in the algae percentage, which increases the accessible functional groups that are suitable for adsorption, hence the increase in adsorption efficiency. Further increase in the algal percentage higher than 20% negatively affected the membrane characteristics, making the membrane brittle. Thus, subsequent experiments were carried out with a 20% algal ratio.

#### 2.1.4. Effect of Dyes Concentration

Different M. blue and C. red concentrations ranging from 50 to 200 ppm were investigated for both types of AF-U and AF-S membranes. Figure S4a,b illustrates that M. blue and C. red removal efficiency rates of around 100% and 90%, respectively, were achieved in the static model at 50 ppm M. blue and C. red concentrations after 12 h contact time. Moreover, it was clear that C. red decreased as concentration increased. This result was similar to those recorded in previous studies, in which pollutant removal decreased by increasing their concentrations [31,32,33]. In contrast, M. blue was nearly constant for both membrane types, i.e., AF-U and AF-S, which indicates the high efficiency of these membranes for removal of higher concentrations of M. blue dye.

### 2.2. Full Factorial Design Experiment (Second Optimization Step)

As a second optimization step, factorial design experiments were performed and analyzed using Minitab 18. The results were interpreted as follows.

#### 2.2.1. Main Effects

**Figure 1:**
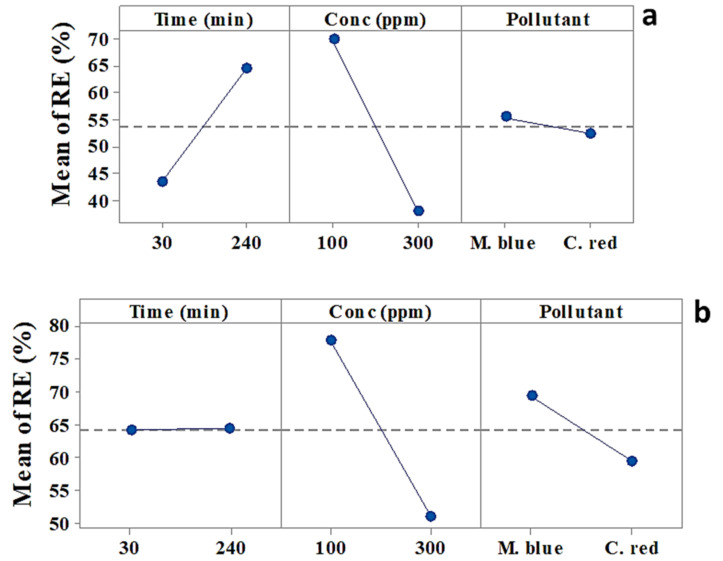
Main effects plot for M. blue and C. red removal efficiency at the low/high levels of each factor (**a**) acrylic fiber *Ulva fasciata* (AF-U) and (**b**) acrylic fiber *Sargassum dentifolium* (AF-S) membranes.

The main effects plotted in Figure 1 show a variation in the average from the low and the high level of each factor, and how this affected the removal efficiency of dyes [34,35]. Figure 1a,b illustrates the main effects of the (a) AF-U and (b) AF-S membranes for both types of dyes, i.e., M. blue and C. red. The removal efficiency of dyes can be noted in the plot when the variation from the low to the high level of a factor increases. This indicates its positive effect on the removal efficiency of the dye. Conversely, removal efficiency decreased as a result of the reduction that occurred from the low to the high level of the factor, which negatively affected the removal efficiency. According to this data, Figure 1a and Table 1 show that only dye concentration had a significant effect and negatively affected the M. blue and C. red removal process using the AF-U membrane. Furthermore, Figure 1a shows that, for the AF-U membrane, 240 min and 100 ppm from dyes achieved the highest removal efficiency, and adsorption of M. blue was greater than that of C. red. By comparison, using AF-S, it can be noted that time had a non-significant effect on the dye removal process (Table 2), and concentration had a significant negative effect on the dye removal process (Table 2 and Figure 1b). Dye removal efficiency decreased by increasing the concentration. Moreover, the pollutant type had a significant effect on the removal process, in which M. blue showed higher removal using the AF-S membrane than C. red. Additionally, the AF-S membrane achieved the highest removal efficiency at 30 min and 100 ppm dye concentration.

#### 2.2.2. Interaction Effect

**Figure 2:**
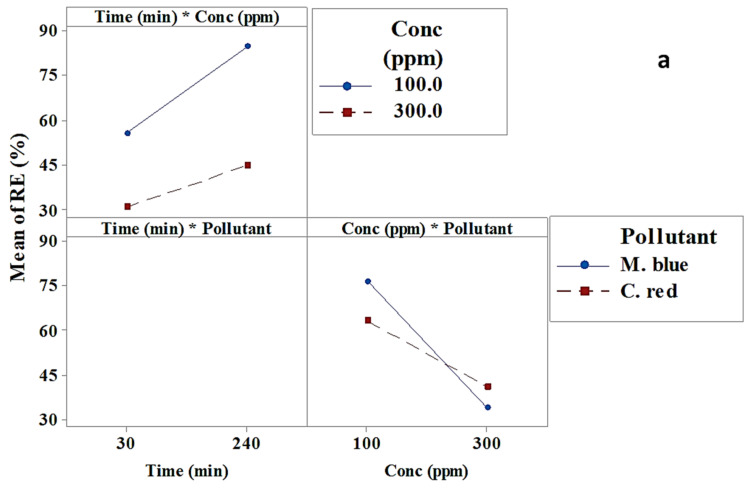

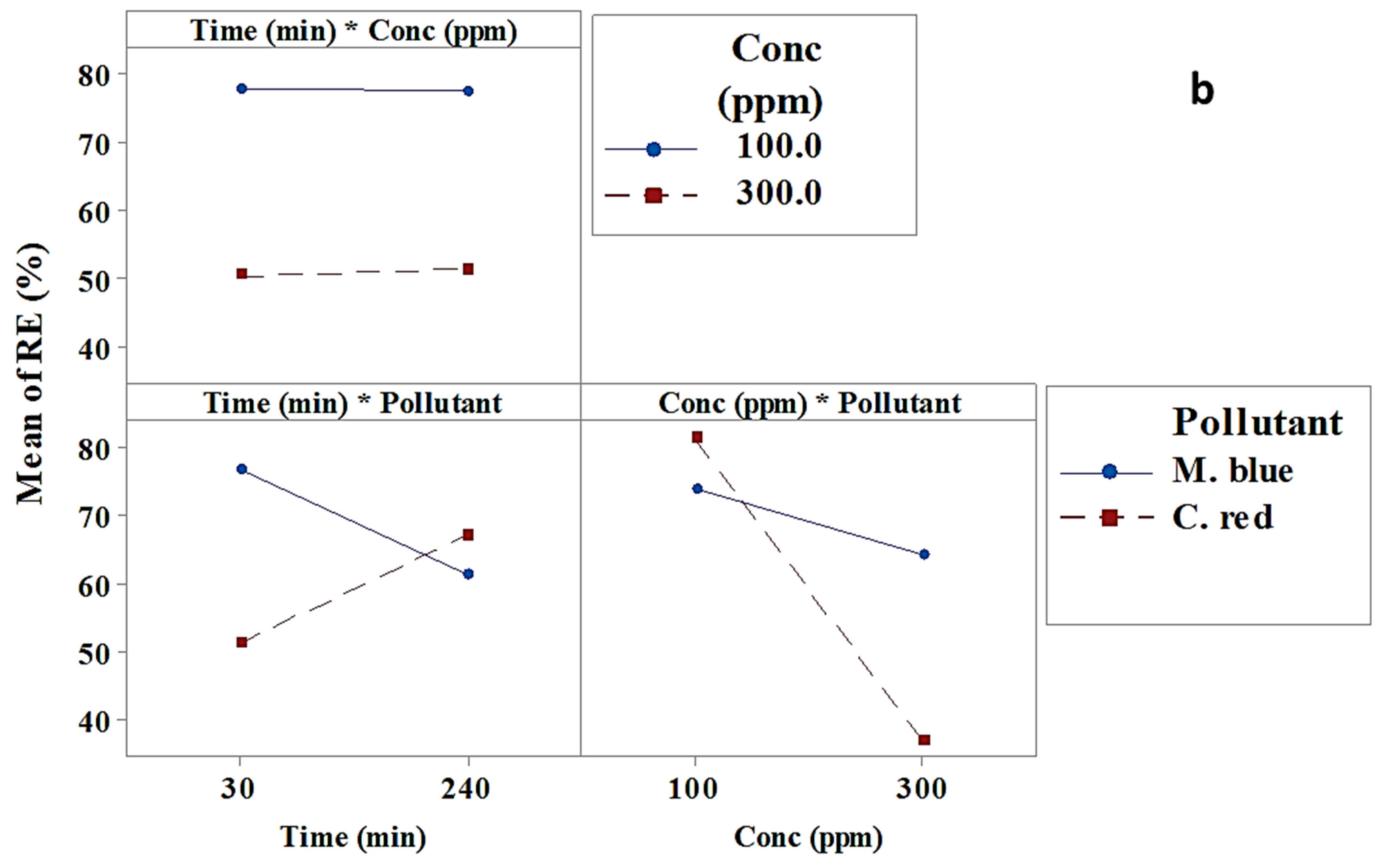
Interaction effects plot for M. blue and C. red removal efficiency at the low/high levels of each factor (**a**) AF-U and (**b**) AF-S membranes.

The interaction effect results from interactions between factors that had an effect on the dye removal efficiency [36]. Figure 2a,b and Table 1 and Table 2 illustrate the interaction effect of optimized factors to obtain the highest dye removal efficiency using AF-U and AF-S. Figure 2a shows that the interaction effect had a non-significant effect on the M. blue and C. red removal process using the AF-U membrane. Nevertheless, Figure 2b and Table 2 show that the interaction between “time and pollutant”, and “conc and pollutant” had a significant effect on the dye removal efficiency using AF-S as an adsorbent membrane.

#### 2.2.3. Pareto Chart

**Figure 3:**
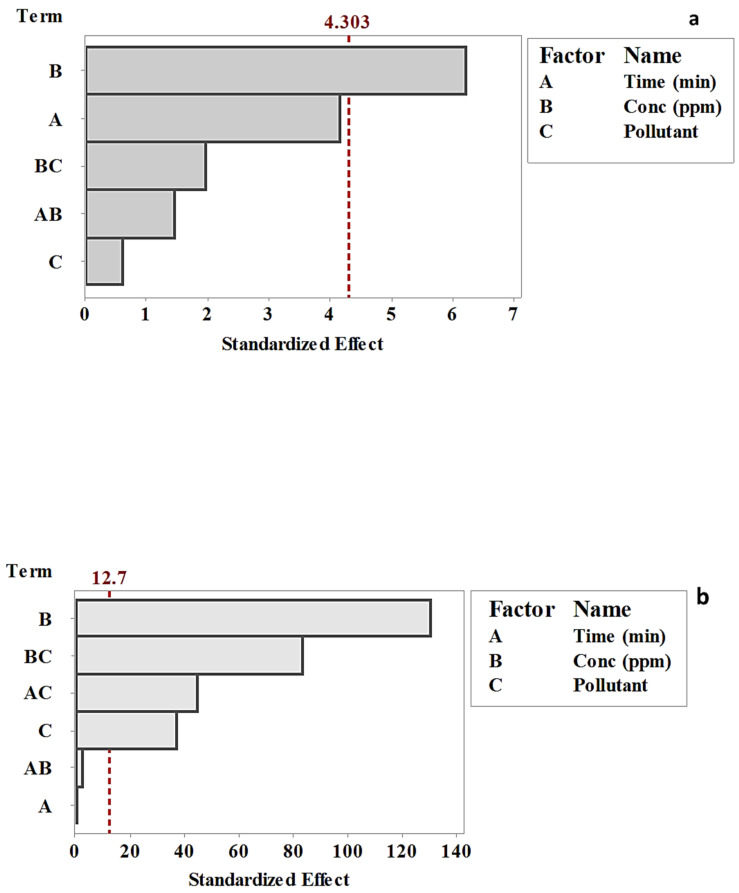
Pareto chart of the standardized main and interaction effects of (**a**) AF-U and (**b**) AF-S membranes.

At 95% confidence intervals and 16 degrees of freedom, the t-value reference line that appears in Figure 3a was 4.303, and in Figure 3b was 12.7, for AF-U and AF-S membranes, respectively [37]. Previously, it was mentioned that all data that exceeded the reference line were significant, whereas the data that did not exceed the reference line were non-significant. Accordingly, only dye concentration (B) was a significant factor that affected the dye removal efficiency using AF-U (Figure 3a). Nevertheless, dye concentration (B), the interaction between concentration and pollutant (BC), the interaction between time and pollutant (AC), and the pollutant type (B) were significant in the adsorption of dyes using the AF-S membrane. These results confirmed the results obtained previously from the main and interaction effects.

#### 2.2.4. Normal Probability Plot

**Figure 4:**
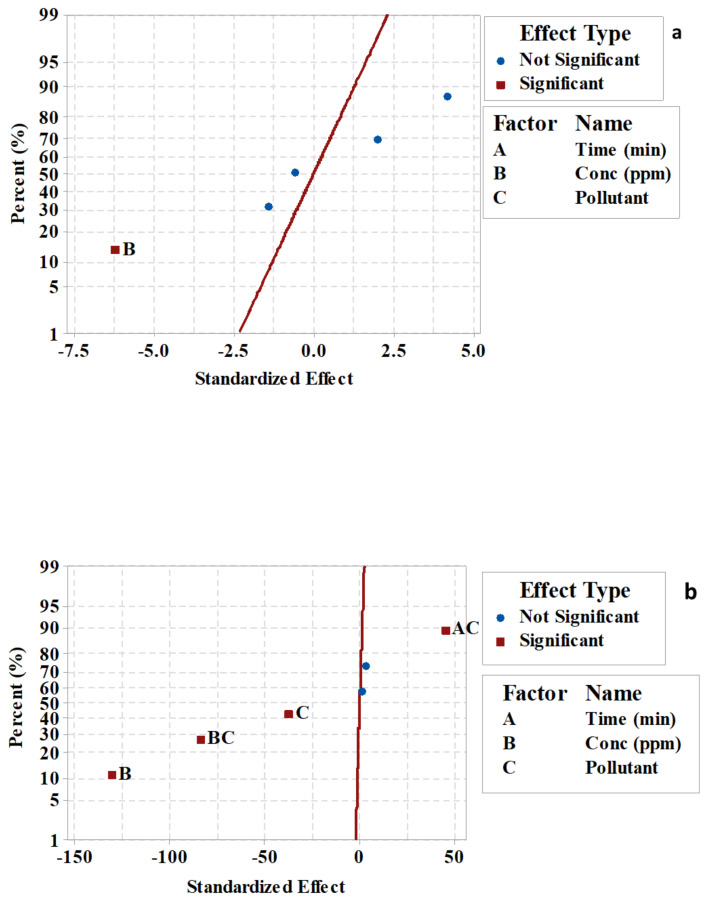
Normal probability plot of standardized main and interaction effects of (**a**) AF-U and (**b**) AF-S membranes.

A normal probability plot was used to determine the real or chance values of the different parameters. Figure 4a,b displays the normal probability plot of AF-U and AF-S membranes for the M. blue and C. red removal process. Each parameter is represented by one point on the plot, which shows that some of the parameters were significant and others were non-significant. Figure 4a shows that only pollutant concentration (B) had a significant effect on the pollutant removal efficiency, due to its location near the line. Nevertheless, Figure 4b shows that pollutant concentration (B), pollutant type (C), the interaction of factor time with pollutant type (AC), and the interaction of the factor concentration with pollutant type (BC) had a significant effect on the removal efficiency process. These results corroborate the previous results obtained from the main and interaction effects, and the Pareto chart.

#### 2.2.5. Response Optimizer

**Figure 5:**
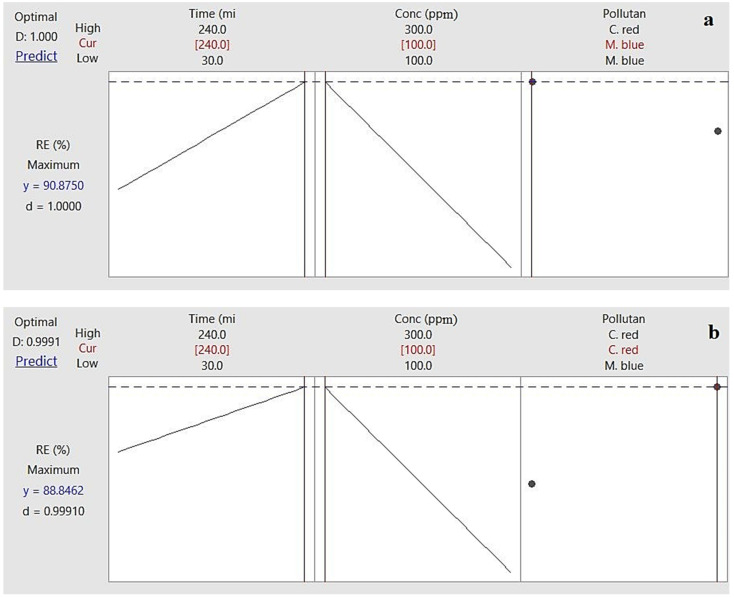
Response optimizer of removal process of (**a**) AF-U and (**b**) AF-S membranes.

Response optimizer is a tool that determines the effect of a group of combined parameters on a response. In this study, the removal efficiency was the response that was investigated against time, pollutant concentration, and pollutant (Figure 5a). M. blue removal efficiency of 90.875% was achieved after 240 min contact time at 100 ppm with a degree of accuracy of 1 using the AF-U membrane. Figure 5b shows that C. red removal efficiency of 88.85% was achieved at 100 ppm after nearly 240 min contact time in a static model with 0.99 degrees of accuracy using the AF-S membrane.

### 2.3. Langmuir and Freundlich Isotherms

**Figure 6:**
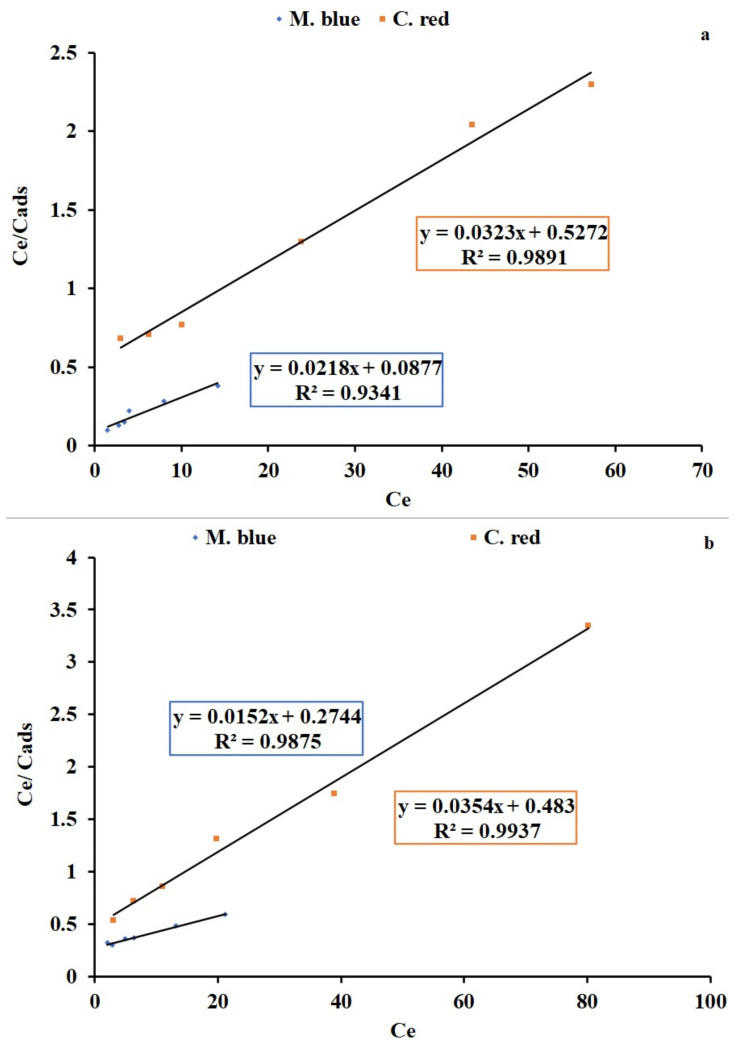
Langmuir isotherm linearized plot for the M. blue and C. red adsorption (**a**) AF-U and (**b**) AF-S membranes.

**Figure 7:**
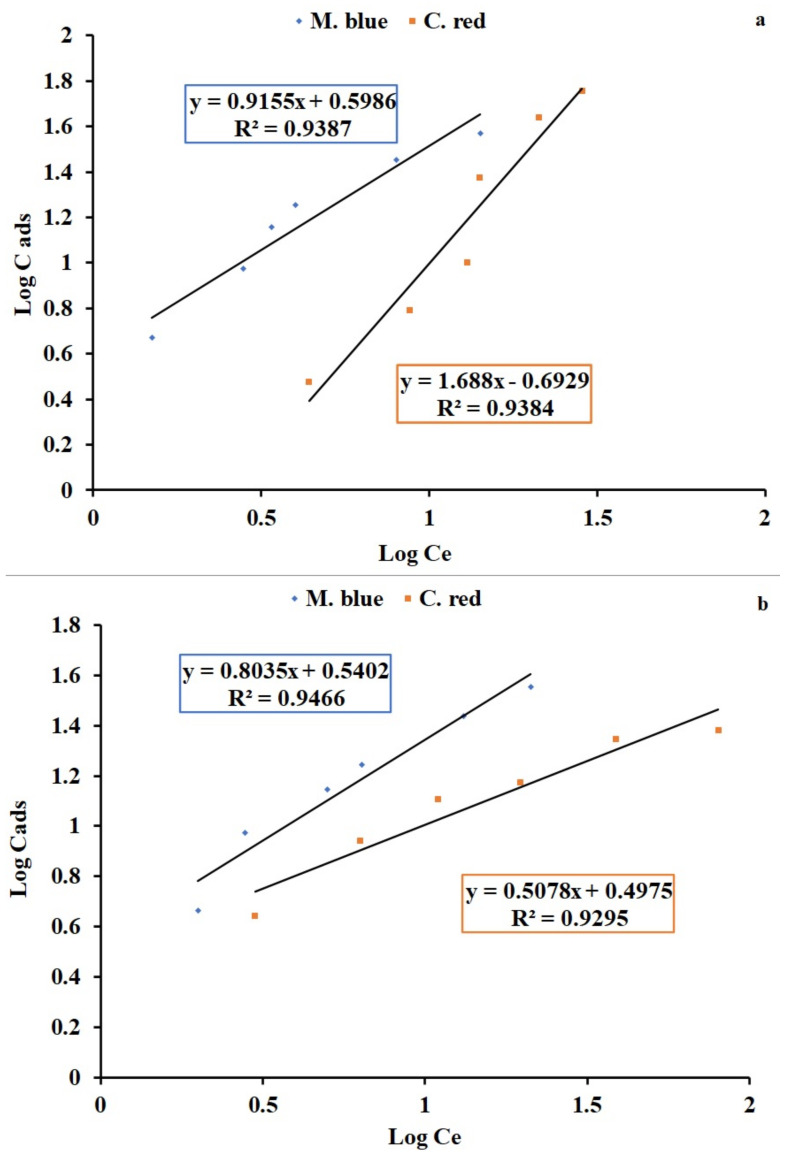
Freundlich isotherm linearized plot for the M. blue and C. red adsorption (**a**) AF-U and (**b**) AF-S membranes.

Adsorption studies were analyzed with Langmuir and Freundlich adsorption isotherm linear equations. The Langmuir isotherm is a linear plot between specific sorption (Ce/qe) and the equilibrium concentration (Ce) of the M. blue and C. red dye adsorption process by applying AF-U and AF-S membranes as adsorbent materials. The linear plots and the calculated results of Langmuir and Freundlich isotherms are displayed in Figure 6a,b and Figure 7a,b for AF-U and AF-S, respectively, and are summarized in Table 3 and Table 4.

Adsorption of M. blue by the AF-U membrane was represented by the Langmuir isotherm model, whereas C. red adsorption by the AF-U membrane matched the Freundlich isotherm model. This can be attributed to the high correlation factor (R^2^) that was noted in both states. Adsorption of M. blue and C. red by AF-S reflected the Langmuir isotherm model. The previous results show that the adsorption nature of the AF-U and AF-S membranes was monolayer adsorption, which means a single layer of M. blue or C. red was formed on the membrane’s surface. Moreover, these results can be attributed to the homogeneous nature of the surface of the AF-U and AF-S membranes. Additionally, it can be suggested that M. blue or C. red dyes were adsorbed completely by the active sites of the AF-U and AF-S membrane surfaces until no further adsorption or interaction could take place. Furthermore, the adsorption capacity, Q_m_, of the two membranes (i.e., AF-U and AF-S) towards the two dyes (M. blue and C. red), were compared with sorbent materials from the previous studies, and are presented in Table S1 [28,37,38,39,40,41,42,43,44].

### 2.4. Kinetics Studies

**Figure 8:**
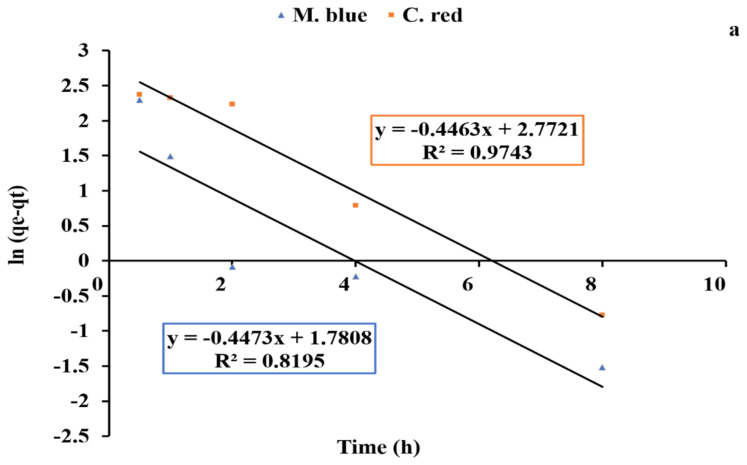

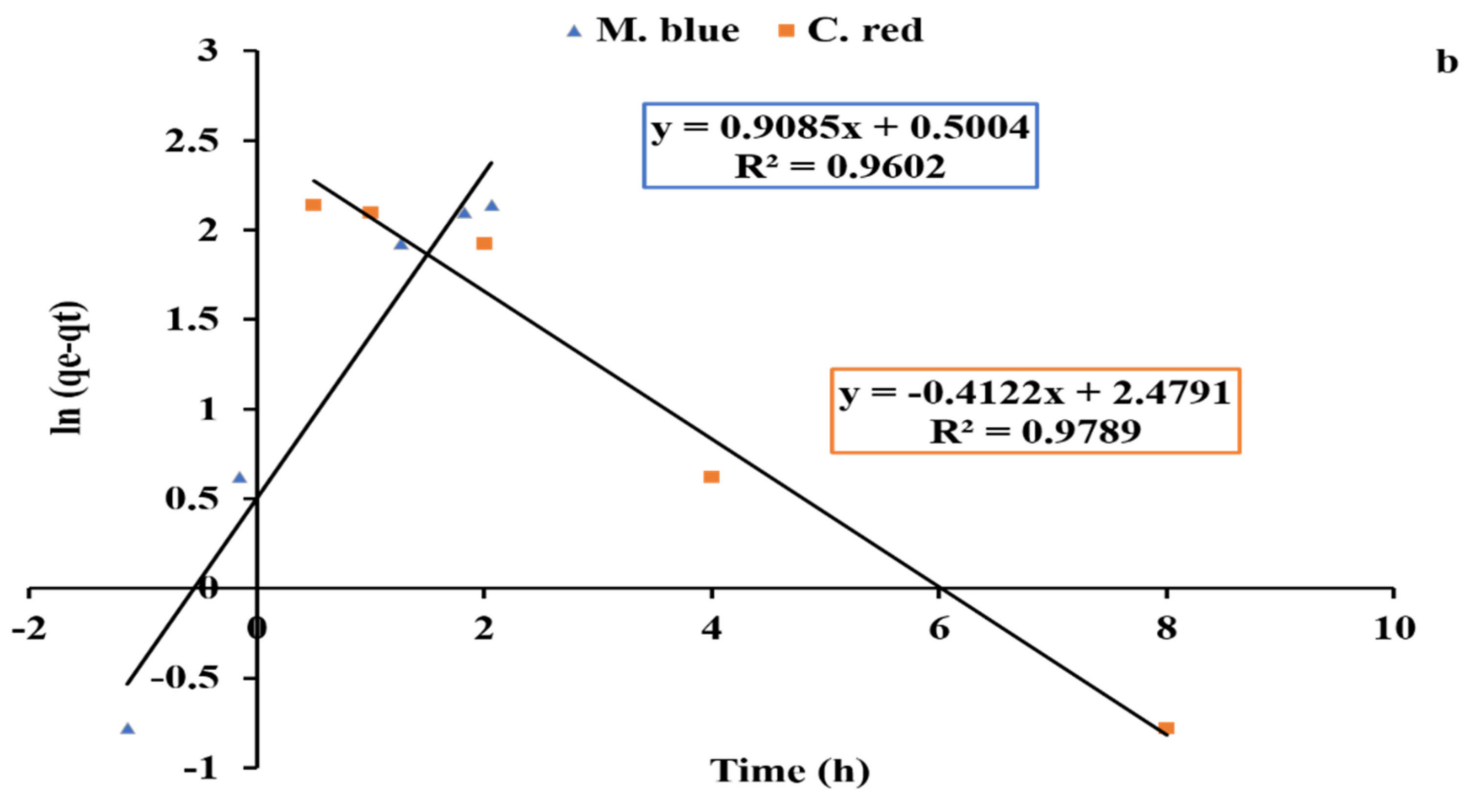
Pseudo first-order reaction of adsorption process of both (**a**) AF- U and (**b**) AF- S membranes.

**Figure 9:**
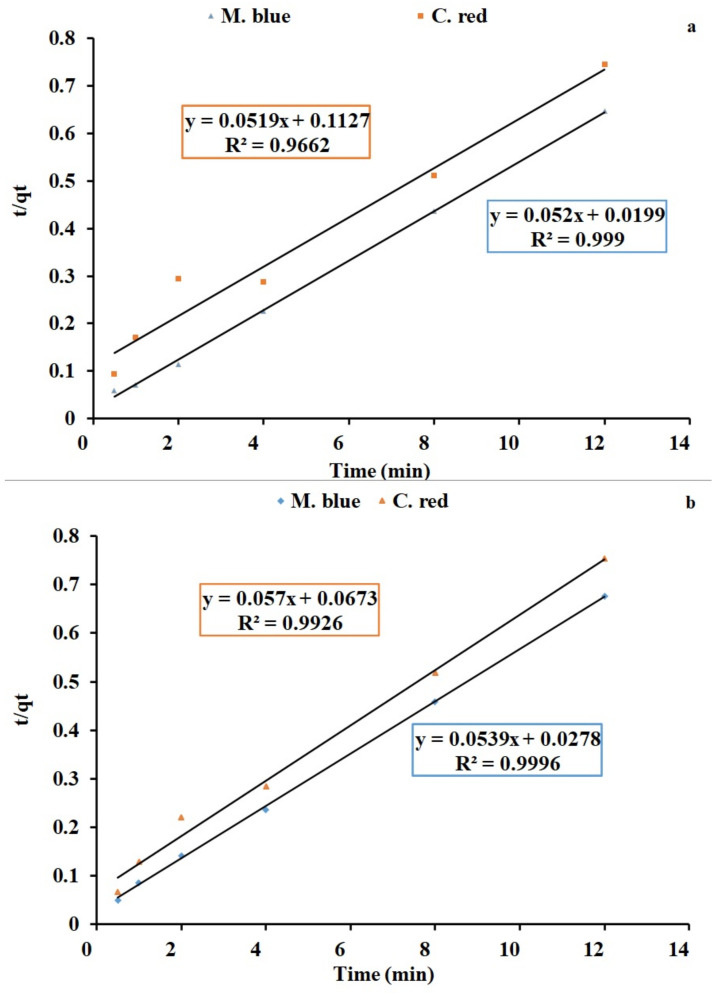
Pseudo second order reaction of adsorption process of (**a**) AF-U and (**b**) AF-S membranes.

Equilibrium kinetics studies are a fundamental analysis technique to evaluate adsorption process affinity and capacity. Pseudo-first-order and second-order linear graphs are shown in Figure 8a,b and Figure 9a,b for AF-U and AF-S membranes, respectively. The kinetics models’ constant values are presented in Table 5 and Table 6. Results of R^2^ indicate that the pseudo second-order model is the model that better describes the adsorption data of the two dyes. Moreover, the calculated result of the pseudo second-order model for q_e_ agrees well with the experimental q_e_ value, unlike the value calculated with the pseudo first-order model. Hence, the second-order kinetics were shown to be the best model that fits with the dye adsorption process. This indicates that the adsorption can be controlled by chemical processes between C. red and AF-U and AF-S membranes [45].

### 2.5. Regeneration Studies

**Figure 10:**
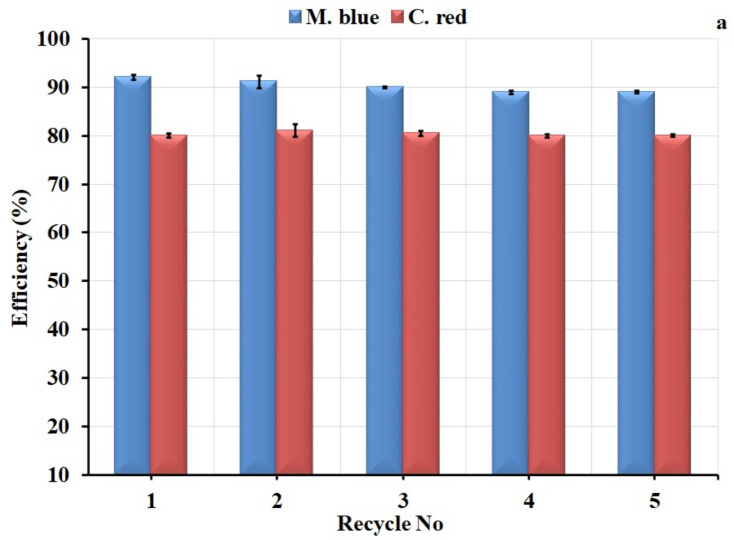

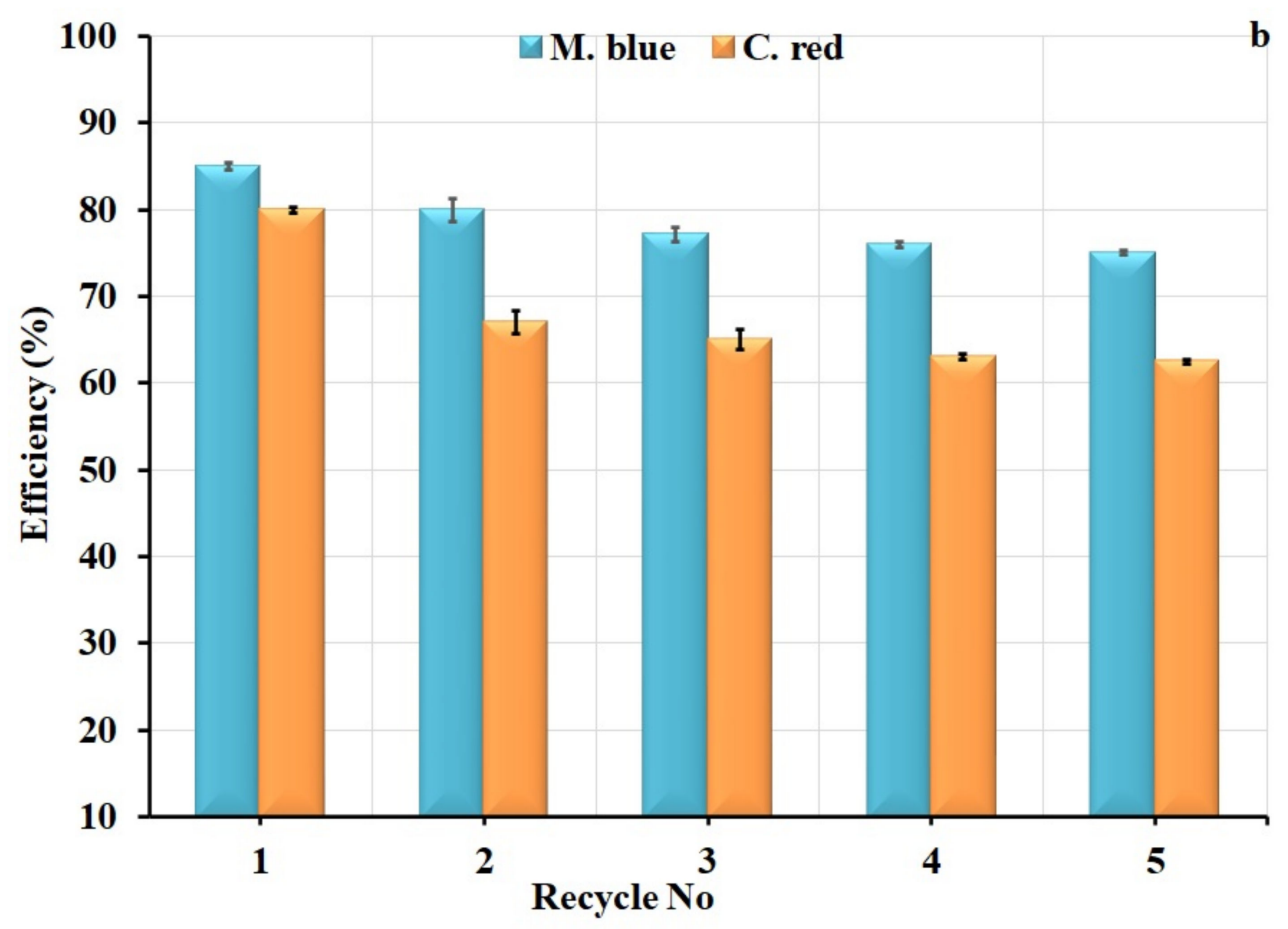
Regeneration study of (**a**) AF-U and (**b**) AF-S membranes.

To assess the stability of algal polymer membranes in the adsorption application, the membranes were used in several adsorption and desorption cycles, as shown in Figure 10. The adsorption efficiency of the AF-U membrane showed a stable performance, declining slightly from 92% to 89% after 5 cycles for M. blue, and displayed a stable reading for C. red of around 80%, as shown in Figure 10a. For the AF-S membrane (Figure 10b), the adsorption decreased from 85% to 75% for M. blue and reduced from 80% to 62.5% for C. red. These results indicate the greater efficiency of the AF-U membrane compared to the AF-S membrane, demonstrating their suitability for the adsorption process during several cycles.

### 2.6. Membrane Characterization

#### 2.6.1. Swelling and Porosity Characteristics of the Membranes

Swelling of the acrylic fiber membrane before and after adding the algal cells was studied; results are recorded in Figure S5. It can be noted that the swelling performance of the membrane increased from nearly 220% to 340% and 320% by adding U. fasciata and S. dentifolium algal membrane, respectively.

The porosity of the membranes was also determined; results are displayed in Figure S6. The porosity of the blank membrane was found to be around 51% from the weight of the polymer membrane and was recorded at around 73% for the AF-U and AF-S membranes. These results may be related to the hydrophilic surface of algal microparticles and the macro-void formation of the membrane in the presence of this algal filler. This clearly appeared in SEM images, which are discussed in the following section.

#### 2.6.2. Fourier Transform Infrared (FTIR) Spectrophotometer

**Figure 11:**
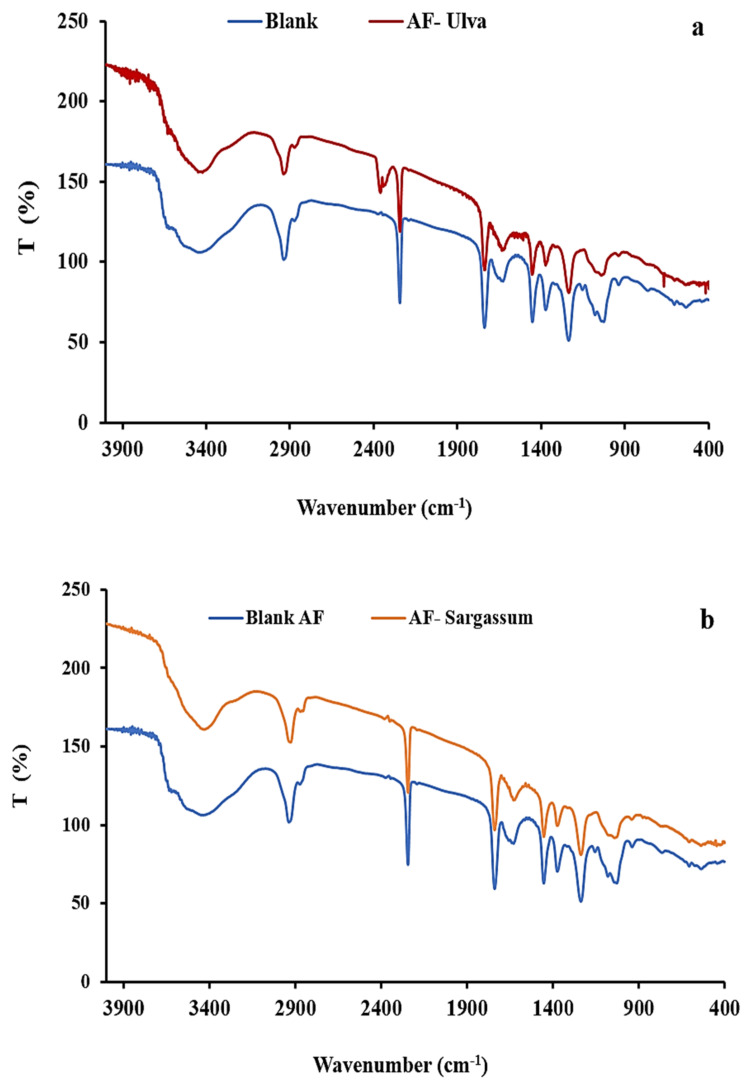
FT-IR of (**a**) AF- U and (**b**) AF- S membranes.

Figure 11a,b shows the FT-IR of the AF membranes with and without algal biomasses. The AF blank membrane had different peaks, first at 2243 cm^−1^, which belongs to CN (nitrile group) of the main polymer [25]; the second peak located at 1738 cm^−1^ corresponds to the C=O group; additionally, the peak at 1450 cm^−1^ of C-O stretching relates to the acrylic fiber additives. Previous research suggested that acrylic fibers comprise at least 85% to 90% acrylonitrile, with the remainder comprising the acrylic-based monomer known as acrylic ester or acid [24]. Moreover, the wide peak at 3418 cm^−1^ could be attributed to absorbed water and the OH group for the additives of the polymer, whereas the peaks at 2860 and 2933 cm^−1^ correspond to the stretching of CH and CH_2_, respectively [46]. The band of 1230 cm^−1^ relates to the bending vibration CH_2_. Furthermore, the IR graph after the incorporation of algal biomasses with polymer, and AF-U and AF-S membrane formation, indicates the intensity of the nitrile group peak located at 2243 cm^−1^ was shortened. Additionally, the wide band around 3420 cm^−1^ was noted to be sharper than the band in the AF blank membrane, which may be attributed to the OH group of the algal particles’ polysaccharide.

#### 2.6.3. SEM Analysis

**Figure 12:**
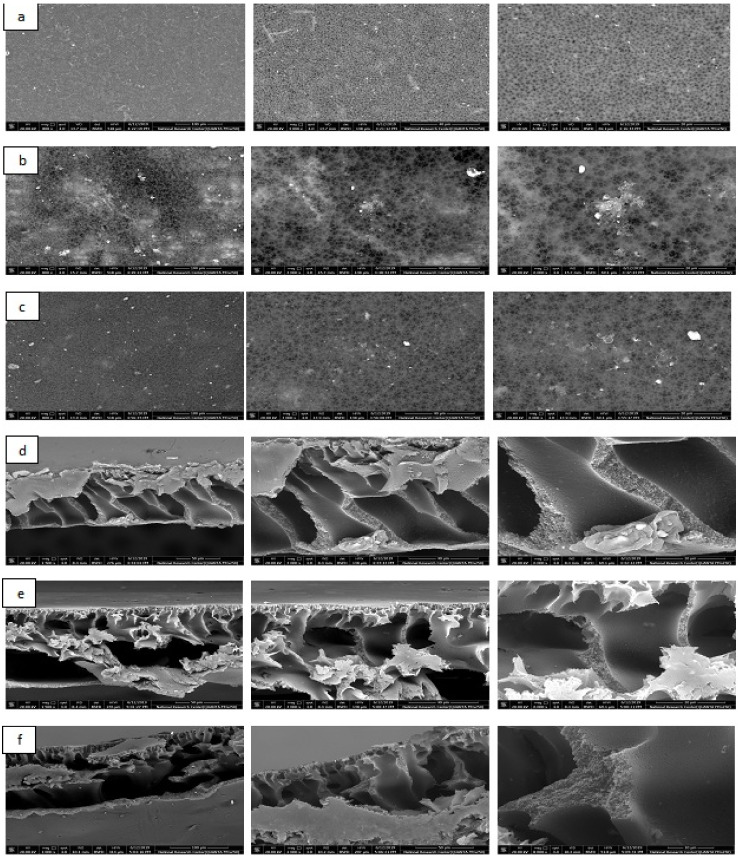
SEM analysis of (**a**) surface of AF blank membrane, (**b**) AF-U, (**c**) AF- S, (**d**) cross section of AF blank membrane, (**e**), cross section of AF- U and (**f**) cross section of AF-S.

The AF membrane surface morphology with and without the incorporation of dried algal biomasses was obtained at different magnifications; results are shown in Figure 12a–e. Figure 12a suggests that the AF blank membrane surface morphology was characterized by a porous structure in which a small fiber-like structure appeared. The previous suggestion was attributed to the inadequate solubilization time. Additionally, the surface of the membrane had small pores that were noted to be distributed on the entire surface. Figure 12b,c shows the surfaces of AF-U and AF-S membranes, respectively, in which their surface is highly porous compared to the AF blank membrane and resembles a sponge-like structure. Moreover, the membrane surfaces appeared to be a multilayer, which has a larger pore size than the AF blank membrane, due to the disappearance of the small fibers. This indicates a well-dissolved process during membrane preparation and the effective sonication step used for distributing the microparticles of algae in the completion of the dissolution of the base polymer. Figure 12d shows that the AF blank membrane cross-section contained a macro-void-like structure with numerous large pores. These indicate the spontaneous de-mixing during the immersion of the membrane solution in the coagulation bath (phase inversion technique) [46,47]. The cross-section of AF-U and AF-S membranes (Figure 12e,f) contains a similar structure with no obvious aggregation of the microparticles of algae at these magnifications.

## 3. Materials and Methods

### 3.1. Algal Biomass Preparation

Two marine macro-algal species *U. fasciata* (Ras El-bar, the Mediterranean Sea) and *S. dentifolium* (Ras gharb, Red Sea) were collected and prepared. After that, the prepared algal biomasses were ball milled to the micro-size by using Planetary Ball Mill, PM 400 “4 grinding stations.

### 3.2. Membrane Preparation

The acrylic membranes with and without marine algal micro-particles were prepared using the phase inversion procedure [7]. Acrylic fibers were dissolved in Dimethylformamide (DMF) solvent under stirring at 60 °C to form 15% polymer solution. After complete dissolution, different ratios of the grinded algae biomass (0, 10, 20, 30% respect to polymer weight) was added to the polymer solution, stirred then sonicated for an hour to confirm well dispersion forming a homogenous mixture. The mixture was casted on a glass plate and spread out by a film applicator with 250 µm thickness. The glass plate was placed in a coagulation water bath; the membranes formed were separated from the plate, left in water for at least 24 h for complete solvent non-solvent exchange, then dried overnight.

#### Pretreatment Method

Chemical treatment of the prepared membrane was performed by a prime solution, which was prepared by mixing 0.5% of “Sodium hydroxide (NaOH)” and 5 % of Sodium chloride (NaCl). The composite membrane was immersed in the prime solution for 1 h for activate the function groups on the embedded algal biomass. After that, the composites membranes were washed with distilled water several times until the pH return to neutral, then left for drying overnight.

### 3.3. Adsorbate Preparation

Textile dyes, 1000 ppm stock solution of M. blue and C. red were obtained by dissolving 1 g of their powder in one liter of distilled water. Serial dilution was prepared from the stock solution according to every experiment.

### 3.4. Batch Sorption Experiments

Different parameters were optimized through two stages optimization, the first stage was studying the effect of each parameter alone “OFAT method” and the second stage was full factorial design experiments. The effects of various parameters on the rate of adsorption process were observed by varying the adsorbent concentration of the solution from 50 to 200 ppm after 12 h contact time, pH 5, and 5 g/L dose by using incorporated algal percentage 20%. Furthermore, pH was ranged from 3–9 by using 50 ppm dyes concentration, 20% algae % and 5 g/L dose after 12 h static contact time. Whereas the algal dose percentage was varied from 5:20% when dose was 5 g/L, and contact time was 12 h static, and finally contact time was changed from ½:12 h at 50 ppm dyes concentration, pH 5, dose of the membrane 5 g/L. All experiments were performed at static model and room temperature ± 25 °C as a two-replicates for each parameter for both of membrane types AF-U and AF-S and applied on the two dyes, M. blue and C. red. After that, the residuals concentration before and after the treatment process were measured using UV-Visible spectrophotometer (Cary 100 UV–Vis) at a wavelength 668 nm for M. blue and 497 nm for C. red percentage of dyes removal or the removal efficiency (RE %) was calculated by the next Equation:

(1) $$\mathrm{Removal}\;\mathrm{efficiency}\;\left(\%\right)=\frac{C_{o}-C_{e}}{C_{o}}\times 100$$

where *C_o_* and *C_e_* are dye concentration at initial and at equilibrium (ppm).

Additionally, adsorption capacity was calculated by using the following Equation:

(2) $$\mathrm{Adsorption}\;\mathrm{capacity}\;(\mathrm{mg}/g)\;=\;\frac{\left(C_{o}-C_{e}\right)\times V}{m}$$

where *C_o_* and *C_e_* are dye concentration at initial and at equilibrium (ppm), V (L) is the volume of the solution, and m (g) is the mass of the membrane.

OFAT experiments were performed in order to determine the low and the high levels of each factor to be included through the factorial design experiment. Data of OFAT experiments were plotted with standard deviation results, where two replicates for each sample were performed. Adsorption capacity data were calculated and presented in Tables S2 and S3.

### 3.5. Full Factorial Design Experiments (Second Optimization Step)

General full factorial design (2^3^) experiments as the second step of optimization were applied for AF-U and AF-S membranes on the two selected dyes to achieve the best conditions that give the highest percentage of removal efficiency. Factorial design table containing the measured dyes removal efficiency, fitted, residual values, and adsorption capacity results were shown in Table 7, Table 8, Table 9 and Table 10. The results were statistically analyzed with the Minitab 18 software and the factorial design plots were plotted and discussed.

### 3.6. Langmuir and Freundlich Isotherm

The linear Langmuir and Freundlich isotherm were studied for the determination of I the sorption of both M. blue and C. red into the two composite membranes, AF-U and AF-S membranes mechanism.

#### 3.6.1. Langmuir Isotherm

The sorption model of Langmuir was designed to investigate the maximum dyes biosorption mechanism and it was expressed through the next Equation [48]

(3) $$\frac{Ce}{Qe}=\frac{Ce}{Qmax}+\frac{1}{bQmax}\;$$

where *b* represented a constant that represent the adsorption/desorption capacity of the AF-U and AF-S membranes, and *Q*_max_ is the highest sorption capacity upon complete saturation of the surface.

#### 3.6.2. Freundlich Isotherm

The sorption model of Freundlich was applied for the purpose of adsorption intensity estimation towards the AF-U and AF-S membranes. After that, the model was expressed by the following Equation: [49]

(4) $$\log Qe=\log Kf+\frac{1}{n}\log Ce$$

where *Q*e is the adsorption density (mg of dye adsorbed/g of AF-U and AF-S membranes; *Ce* = concentration of dye molecules in solution at equilibrium (ppm); *K*_f_ and *n* are the Freundlich constants. See Table 7 and Table 8.

### 3.7. Kinetics Studies

Adsorption process kinetics issued to the rate of composite membranes; AF-U and AF-S adsorption for M. blue and C. red dyes, which control the equilibrium time. Kinetics models that include the first-order and second-order Equations were indicated as follows:

#### 3.7.1. Pseudo First-Order Model

The model of the pseudo first order was described by the following Equation [50]

(5) $$\log\left(qe-qt\right)=\log qe-\;\frac{K1t}{2.303}$$

where *q*_e_ is adsorbed dyes at equilibrium per unit weight of AF-U and AF-S membranes (mg/g), *q*_t_ is adsorbed dyes at a time *t* (mg/g), and *k*_1_ is the rate constant (min).

#### 3.7.2. Pseudo Second-Order Model

The data were analyzed by the pseudo second-order Equation [51]

(6) $$\frac{t}{q_{t}}=\frac{1}{K_{2}q_{e}{}^{2}}+\frac{t}{q_{e}}$$

where *K*_2_ is the equilibrium rate constant (g/mg min), and *q*_e_ and *q*_t_ are the sorption capacities of the AF-U and AF-S membranes at an equilibrium and at a time *t*, respectively.

### 3.8. Characterization of the Composite Membranes

#### 3.8.1. Swelling Properties

The AF blank, AF-U, and AF-S membranes swelling properties were investigated by immersing known weight of acrylic fiber membrane in distilled water at ±25 °C in atmospheric conditions for a day to reach swelling equilibrium [52]. After that, the membrane was pulled out from the water and specked with filter paper and weighed. The capacity of swelling was measured by the following Equation:

(7) $$Swelling\left(\%\right)=\frac{W_{2}-W_{1}}{W_{1}}\times 100$$

where *W*_1_ (g) weights of the dried AF-U and AF-S membranes and *W*_2_ (g) are the weights of the swollen AF-U and AF-S membranes.

#### 3.8.2. Porosity Properties

The porosity of the AF blank, AF-U, and AF-S membranes were estimated by the dry–wet weight method [53,54]. The wet weight of the composite membranes with definite dimension was demonstrated after discarding of the excess water. Afterwards, the wet AF-U and AF-S membranes were dried in a vacuum oven at 60 °C for 24 h and the dry weight was determined. Finally, the AF-U and AF-S membranes porosity were calculated as follow:

(8) $$\varepsilon \;\left(\%\right)=\left[\frac{\mathrm{Wwet}\;-\mathrm{Wdr}}{\mathrm{dwAh}}\right]\;\times \;100$$

where ε is the porosity of the AF-U and AF-S membranes, d_w_ is the density of pure water (0.998 g/cm^3^), A is the AF-U and AF-S membranes area in the wet state (cm^2^), and h is the thickness of composite AF-U and AF-S membranes in the wet state (cm).

#### 3.8.3. Fourier Transform Infrared Spectroscopy (FT-IR)

FT-IR analysis were displayed to detect the functional groups of the AF-U and AF-S membranes within a range of 400–4000 cm^−1^ in a (Shimadzu) spectrophotometer.

#### 3.8.4. Scanning Electron Microscopy (SEM)

The morphology of the AF-U and AF-S membranes surface and the cross-sections were investigated using Scanning Electron Microscopy (SEM). Before the examination, the membranes were sputter-coated with gold. The SEM micrographs were demonstrated at an accelerating voltage of 15 kV (Hitachi SE 900) at different magnification powers.

### 3.9. Desorption Studies

The stability and reusability of the prepared composite membranes; the AF-U and AF-S membranes evaluation were soaked in 50% aqueous ethanol solution to remove the adsorbed dyes. This step was repeated twice to discard the remained dyes that were cleaned with distilled water after that and left for drying at ambient temperature for a day. The dried membranes were reused in another adsorption cycle and this process was repeated for many cycles.

## 4. Conclusions

Preparation of adsorptive membranes based on acrylic fiber waste with *Ulva fasciata* and *Sargassum dentifolium* biomasses was investigated and applied to dye removal from aqueous solutions with an easily handled process and high efficiency. The use of AF-U and AF-S membranes in the Methylene blue and Congo-red removal process resulted in high removal efficiency for the two types of dyes. For the OFAT optimization, the Methylene blue and Congo red removal efficiency rates were around 100% and 90%, respectively, at 50 ppm concentrations after 12 h contact time in a static model. Furthermore, by applying factorial design experiments, AF-U showed Methylene blue removal of 88.9% and Congo red removal of 79.6% after 240 min contact time at 100 ppm concentration. Moreover, AF-S achieved Methylene blue removal of 82.01% after 30 min contact time and Congo red removal of 88.9% after 240 min contact time, at 100 ppm concentration in a static model. From these results it can be concluded that AF-U had higher removal efficiency for Methylene blue and Congo red than AF-S. Furthermore, factorial design helped achieve higher Methylene blue and Congo red removal efficiency than OFAT when the contact time was shorter. Additionally, the static model was a promising tool for the removal process with a lower cost than the shaking model. AF-U and AF-S were successfully applied to the 3Rs process (i.e., removal, recovery, and re-use). In future investigation, more polymer materials can be investigated for incorporation with algal biomasses and more types of algae can be examined. This work demonstrated wastewater treatment using natural waste (algae), which can be used without the need for a physical-chemical modification process. This material is ecofriendly and does not produce any harmful byproducts. This further enhances the importance of using algae in these applications, demonstrating their significant advantages, and making them attractive for application in this field of research.

## Figures and Tables

**Table 1:** Coded Co-efficient for M. blue and C. red adsorption by using AF-U.

Term	Effect	Coef	SE Coef	T-Value	*p*-Value
Constant		53.80	2.58	20.89	0.002
Time (min)	21.42	10.71	2.58	4.16	0.053
Conc (ppm)	−32.05	−16.03	2.58	−6.22	0.025
Pollutant	−3.10	−1.55	2.58	−0.60	0.608
Time (min) × Conc (ppm)	−7.43	−3.72	2.58	−1.44	0.286
Conc (ppm) × Pollutant	10.15	5.08	2.58	1.97	0.188

**Table 2:** Coded Co-efficient for M. blue and C. red adsorption by using AF-S membrane.

Term	Effect	Coef	SE Coef	T-Value	*p*-Value
Constant		64.192	0.133	483.69	0.001
Time (min)	0.303	0.152	0.173	0.88	0.542
Conc (ppm)	−26.851	−13.425	0.103	−130.34	0.005
Pollutant	−9.933	−4.967	0.133	−37.42	0.017
Time (min) × Conc (ppm)	0.698	0.349	0.126	2.78	0.220
Time (min) × Pollutant	15.587	7.793	0.173	44.96	0.014
Conc (ppm) × Pollutant	−17.199	−8.600	0.103	−83.49	0.008

**Table 3:** Langmuir and Freundlich Isotherm parameters for M. blue and C. red adsorption by the AF-U membrane.

Model	Parameters					
Langmuir	Q_max_ (mg/g)		B		R^2^	
	M. blue	C. red	M. blue	C. red	M. blue	C. red
	45.871	30.959	0.2485	0.0408	0.9341	0.9891
Freundlich	n		K		R^2^	
	M. blue	C. red	M. blue	C. red	M. blue	C. red
	1.0922	0.5924	3.9682	4.9306	0.9387	0.9384

R^2^, correlation coefficient. M. blue, Methylene blue. C. red, Congo red.

**Table 4:** Langmuir and Freundlich Isotherm parameters for M. blue and C. red adsorption by the AF-S membrane.

Model	Parameters					
Langmuir	Q_max_ b				B	R^2^
	M. blue	C. red	M. blue	C. red	M. blue	C. red
	65.789	28.24	0.0553	0.073	0.9875	0.9937
Freundlich	n		K		R^2^	
	M. blue	C. red	M. blue	C. red	M. blue	C. red
	1.244	1.969	3.4689	3.14412	0.9466	0.9295

R^2^, correlation coefficient. M. blue, Methylene blue. C. red, Congo red.

**Table 5:** Kinetic parameters for M. blue and C. red adsorption by the AF-U membrane. pH value: 7, initial concentration: 50 ppm, contact time: ½:15 h, at static model.

Model	Parameters							
1st order Kinetics	q_e_ calc.		1st order Kinetics		q_e_ calc.		1st order Kinetics	
	M. blue	C. red	M. blue	C. red	M. blue	C. red	M. blue	C. red
	60.3	778.7	60.3	16.1	60.3	1.07	60.3	0.96
2nd order kinetics	qe calc.		2nd order kinetics		qe calc.		2nd order kinetics	
	M. blue	C. red	M. blue	C. red	M. blue	C. red	M. blue	C. red
	19.23	19.26	19.23	16.1	19.23	0.023	19.23	0.9926

**Table 6:** Kinetic parameters for M. blue and C. red adsorption by the AF-S membrane. pH value: 7, initial concentration: 50 ppm, contact time: ½:15 h, at static model.

Model	Parameters							
1st order Kinetics	q_e_ calc.		1st order Kinetics		q_e_ calc.		1st order Kinetics	
	M. blue	C. red	M. blue	C. red	M. blue	C. red	M. blue	C. red
	301.23	3.16	301.23	16.1	301.23	−2.09	301.23	0.9602
2nd order kinetics	qe calc.		2nd order kinetics		qe calc.		2nd order kinetics	
	M. blue	C. red	M. blue	C. red	M. blue	C. red	M. blue	C. red
	18.5529	17.5439	18.5529	16.1	18.5529	0.04828	18.5529	0.9926

**Table 7:** Low and high levels of 23 full factorial design experiment for M. blue and C. red adsorption by using AF-U sheet.

Factor	Unit	Symbol	Statistical Code	Values of Coded Levels	
				(Low) − 1	(High) + 1
Time	min	Time	A	30	240
Dyes Concentration	ppm	Conc	B	100	300
Pollutant		Pollutant	C	M. blue	C. red

**Table 8:** Low and high levels of 2^3^ full factorial design experiment for M. blue and C. red adsorption by using AF-S sheets.

Factor	Unit	Symbol	Statistical Code	Values of Coded Levels	
				(Low) − 1	(High) + 1
Time	min	Time	A	30	240
Dyes Concentration	ppm	Conc	B	100	300
Pollutant		Pollutant	C	M. blue	C. red

**Table 9:** The design of matrix for M. blue and C. red adsorption by using AF-U.

StdOrder	RunOrder	PtType	Blocks	Time (min)	Conc (ppm)	Pollutant	RE (%)	FITS1	RESI1
4	1	1	1	240	300	M. blue	46	41.24167	4.758333
2	2	1	1	30	300	M. blue	22.5	27.25833	−4.75833
1	3	1	1	30	100	M. blue	64	62.025	1.975
3	4	1	1	240	100	M. blue	88.9	90.875	−1.975
4	5	1	1	240	300	C. red	43.53333	48.29167	−4.75833
1	6	1	1	30	100	C. red	46.8	48.775	−1.975
2	7	1	1	30	300	C. red	39.06667	34.30833	4.758333
3	8	1	1	240	100	C. red	79.6	77.625	1.975

S, 7.285, R-sq, 96.89%, R-sq (adj), 89.11%.

**Table 10:** The design of matrix for M. blue and C. red adsorption by using AF-S.

StdOrder	RunOrder	PtType	Blocks	Time (min)	Conc (ppm)	Pollutant	RE (%)	FITS1	RESI1
3	1	1	1	240	100	M. blue	75	75.12552	−0.12552
4	2	1	1	240	300	M. blue	65.5	65.37448	0.125517
1	3	1	1	30	100	M. blue	82.1	81.97448	0.125517
2	4	1	1	30	300	M. blue	71.5	71.62552	−0.12552
4	5	1	1	240	300	C. red	45.44	45.49379	−0.05379
**3**	6	1	1	240	100	C. red	88.9	88.84621	0.053793
**2**	7	1	1	30	300	C. red	28.96	28.90621	0.053793
**1**	8	1	1	30	100	C. red	73.6	73.65379	−0.05379

S, 0.2731, R-sq, 100%, R-sq (adj), 99.98%.

## Data Availability

Not applicable.

## Supplementary Materials

The following are available online at https://www.mdpi.com/2223-7747/10/2/384/s1, Figure S1. Effect of contact time on M. blue and C. red adsorption by (a) AF- U and (b) AF- S membranes, Figure S2. Effect of pH on M. blue and C. red adsorption by (a) AF- U and (b) AF- S membranes, Figure S3. Effect of dose on M. blue and C. red adsorption by (a) AF- U and (b) AF- S membranes, Figure S4. Effect of M. blue and C. red concentration on their adsorption process by (a) AF- U and (b) AF- S membranes, Figure S6. Porosity characteristics of AF blank, AF-U, AF-S membranes. Table S1: Adsorption capacity of AF-U membrane for both of M. blue and C. red, Table S2: Adsorption capacity of AF-S membrane for both of M. blue and C. red, Table S3: Comparison of M. blue and C. red dyes removal by different sorption materials.
