# Supplementary material for: Removal of Methylene Blue and Congo Red Using Adsorptive Membrane Impregnated with Dried Ulva fasciata and Sargassum dentifolium
Source: Plants (Basel). 2021 Feb 17;10(2):384. doi: 10.3390/plants10020384 (PMC7922868; doi:10.3390/plants10020384)
Supplement: Supplementary file 1 [file plants-10-00384-s001.pdf]

plants

MDPI

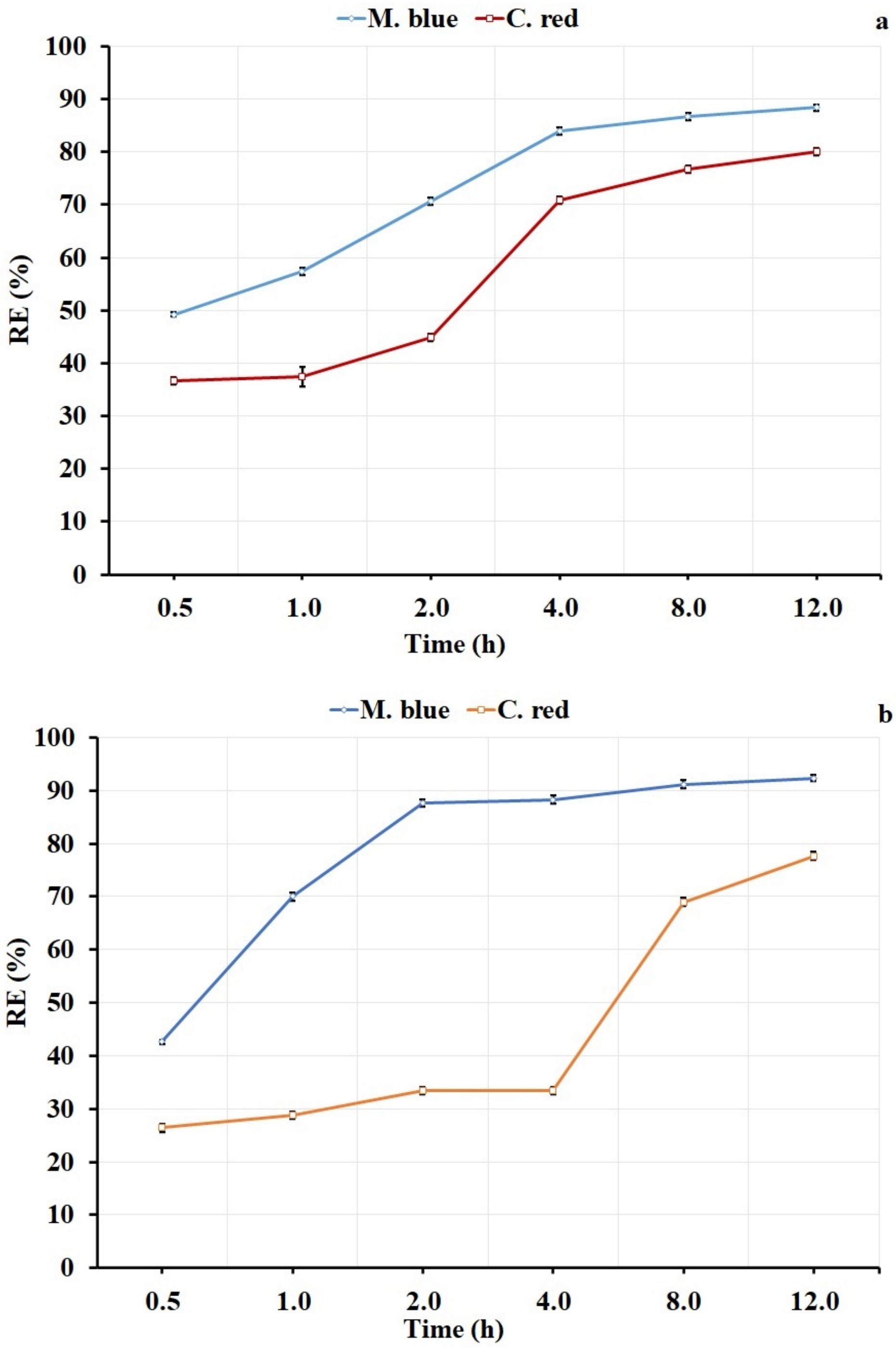

Figure S1**.** Effect of contact time on M. blue and C. red adsorption by (a) AF- U and (b) AF- S membranes.

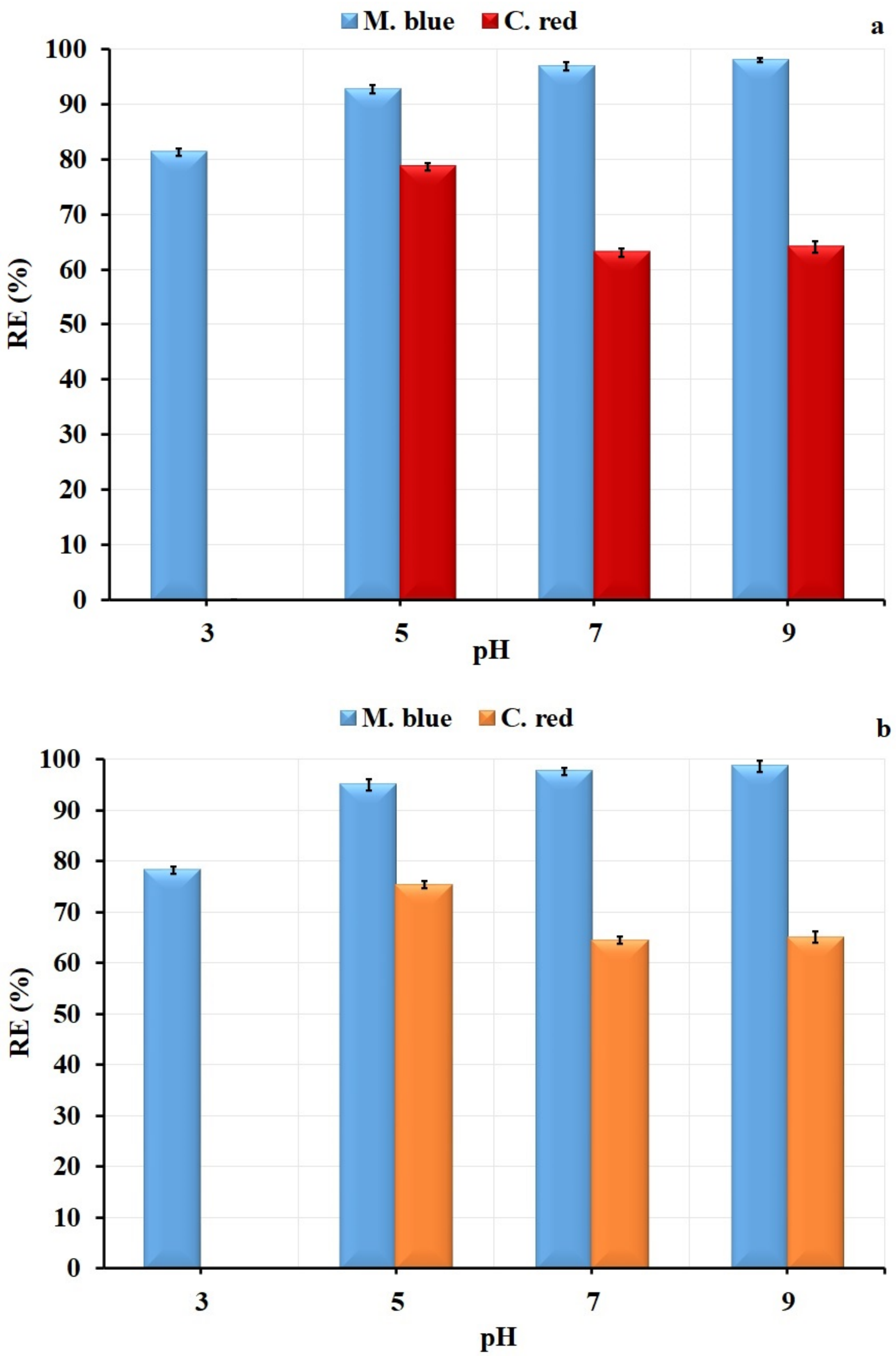

Figure S2. Effect of pH on M. blue and C. red adsorption by (a) AF- U and (b) AF- S membranes.

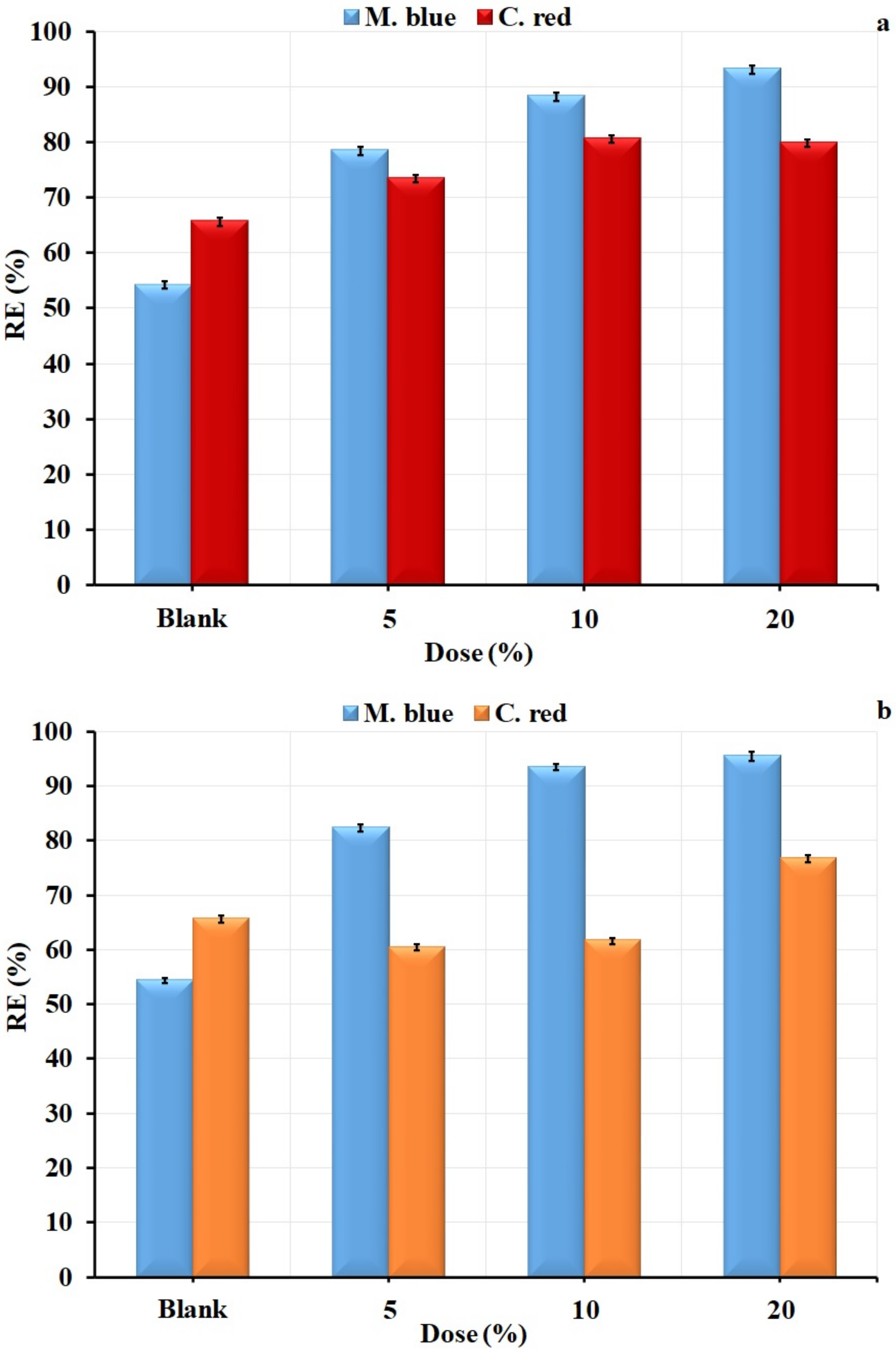

M. blue
C. red
a
RE (%)
100
90
80
70
60
50
40
30
20
10
0
Blank
5
10
20
Dose (%)
M. blue
C. red
b
RE (%)
Dose (%)

Figure S3. Effect of dose on M. blue and C. red adsorption by (a) AF- U and (b) AF- S membranes.

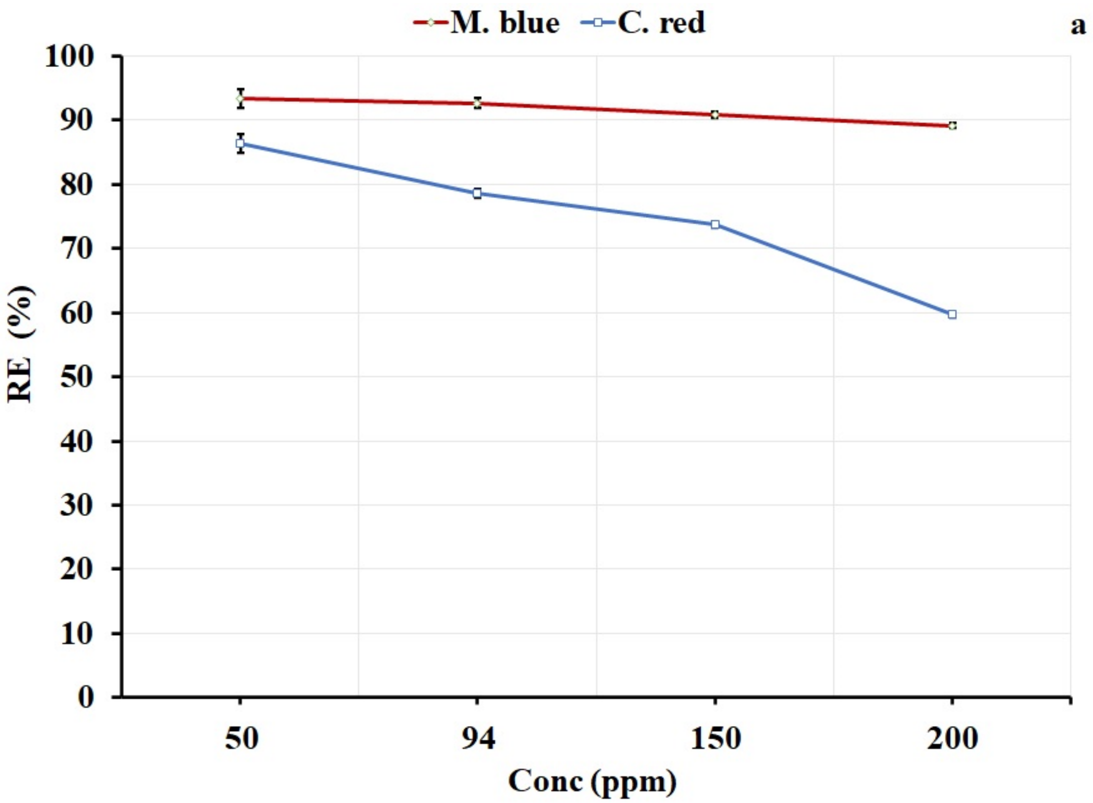

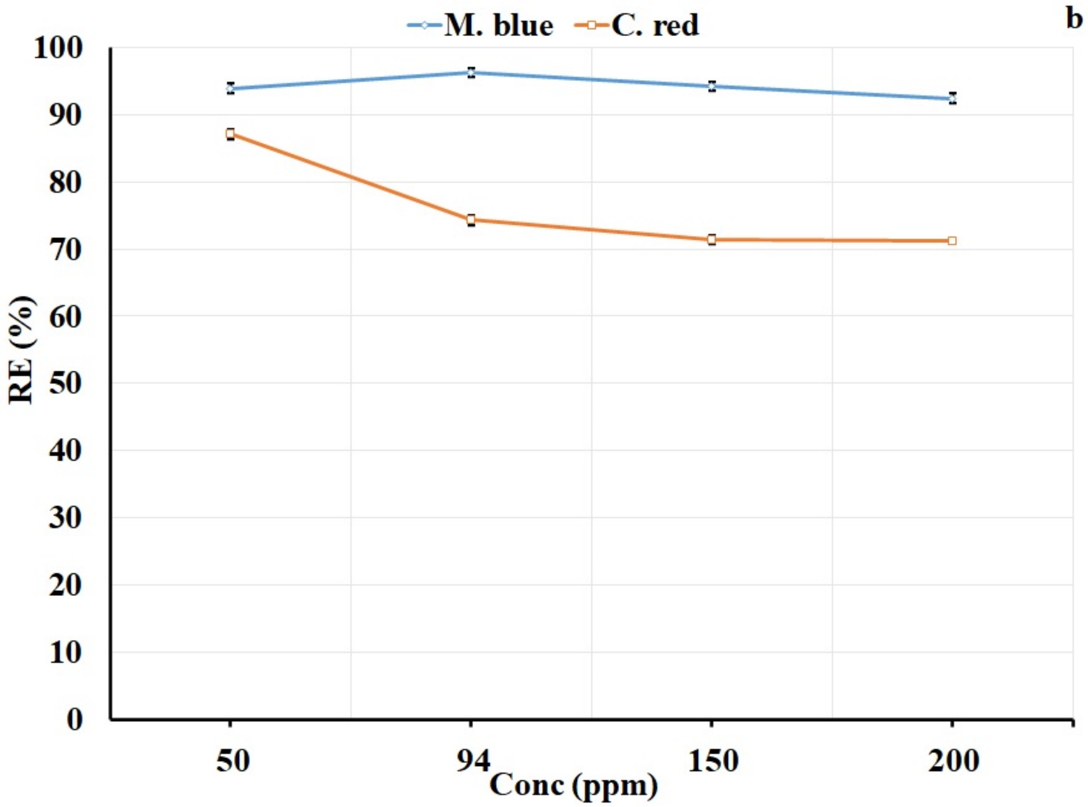

Figure S4. Effect of M. blue and C. red concentration on their adsorption process by (a) AF- U and (b) AF- S membranes.

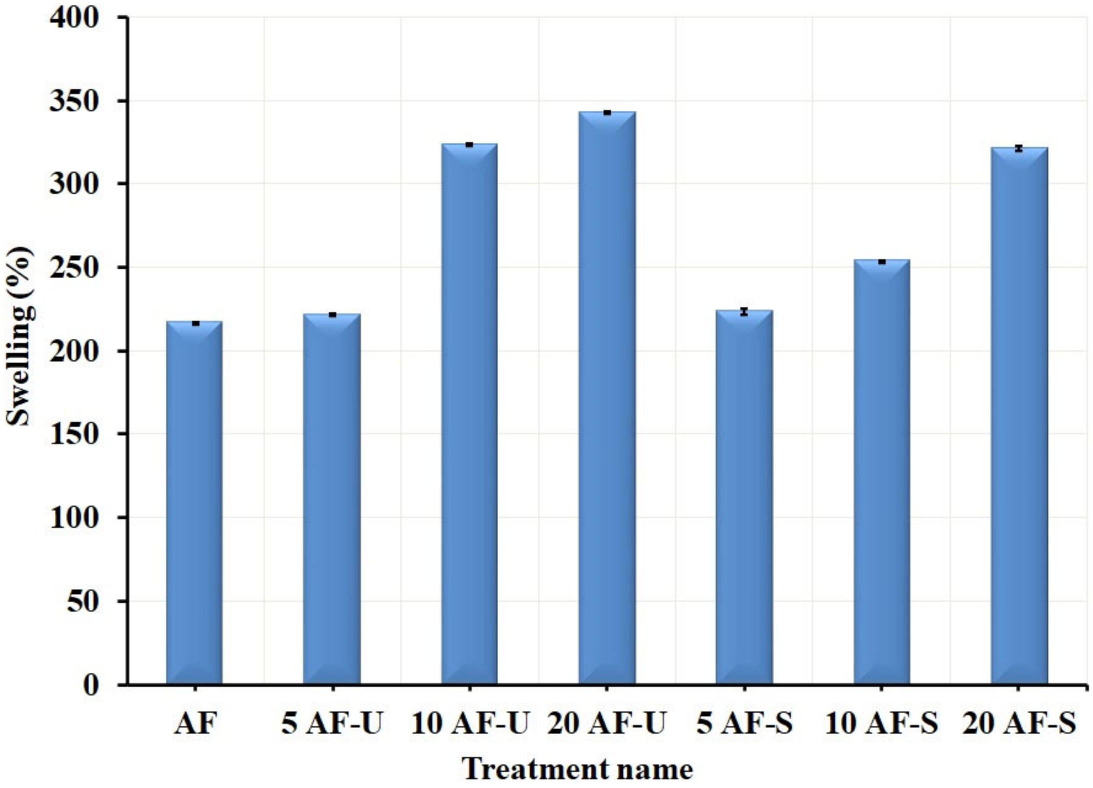

Figure S5. Swelling characteristics of AF blank, AF-U, AF-S membranes.

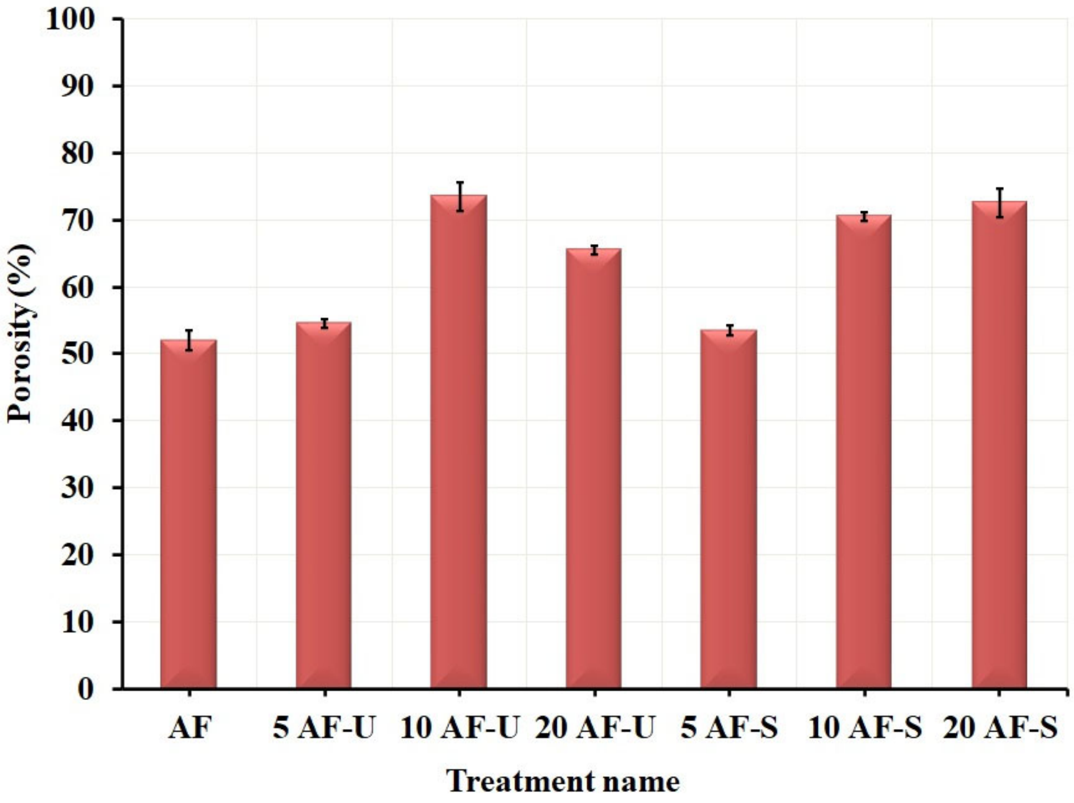

Figure S6. Porosity characteristics of AF blank, AF-U, AF-S membranes.

Table S1: Adsorption capacity of AF-U membrane for both of M. blue and C. red

Treat	time (h)	pH	Dose (%)	Conc. (ppm)	M. blue Qe (mg/g)	C. red Qe (mg/g)
AF-U	½	7	20	100	8.62	5.38
AF-U	1	7	20	100	14.1	5.86
AF-U	2	7	20	100	17.64	6.78
AF-U	4	7	20	100	17.76	13.9
AF-U	8	7	20	100	18.34	15.64
AF-U	12	7	20	100	18.56	16.1
AF-U	12	3	20	94	14.8	-
AF-U	12	5	20	94	18	14.14
AF-U	12	7	20	94	18.46	12.28
AF-U	12	9	20	94	18.6	12.9

AF-U	12	7	Blank	100	10.94	13.22
AF-U	12	7	5	100	16.36	12.16
AF-U	12	7	10	100	18.6	12.24
AF-U	12	7	20	100	19.2	15.24
AF-U	12	7	20	50	9.44	8.76
AF-U	12	7	20	94	18	14.14
AF-U	12	7	20	150	28.4	21.3
AF-U	12	7	20	200	37.16	28.56

Table S2: Adsorption capacity of AF-S membrane for both of M. blue and C. red

Treat	time	pH	Dose (%)	Conc.	M. blue Q_e (mg/g)	C. red Q_e (mg/g)
AF-S	1/2	7	20	100	9.9	12.58
AF-S	1	7	20	100	11.58	12.24
AF-S	2	7	20	100	14.22	10.92
AF-S	4	7	20	100	16.9	5.94
AF-S	8	7	20	100	17.44	4.54
AF-S	12	7	20	100	17.76	4.08
AF-S	12	3	20	94	15.38	-
AF-S	12	5	20	94	17.52	14.96
AF-S	12	7	20	94	18.3	12.02
AF-S	12	9	20	94	18.52	12.5
AF-S	12	7	Blank	100	10.94	13.22
AF-S	12	7	5	100	15.78	14.78
AF-S	12	7	10	100	17.74	16.22
AF-S	12	7	20	100	18.72	16.06
AF-S	12	7	20	50	8.112	8.74
AF-S	12	7	20	94	15.2	14.96
AF-S	12	7	20	150	24.32	22.22
AF-S	12	7	20	200	32.568	23.96

Table S3: Comparison of M. blue and C. red dyes removal by different sorption materials.

Adsorption material	Dye	Conc (ppm)	Adsorption capacity (Qe mg/g)	References
**AF-U membrane**	M. blue	50-200	63.59	Current study
**AF-U membrane**	C. red	50-200	39.84	Current study
**AF-S membrane**	M. blue	50-200	62.5	Current study
**AF-S membrane**	C. red	50-200	28.57	Current study
**Defatted algal biomass**	M. blue	1–5	7.8	[38]
**Eichhornia crassipes biomass**	C. red	3:06	14.49	[38]
**CB-CTAB**	C. red	20	19.15	[39]
**Pinus brutia**	C. red		35.3	[40]
**U. reticulata**	C. red	5:35	42.3	[41]
**S. crassifolium**	C. red	5:35	43.4	[41]
**G. corticate**	C. red	5:35	41.22	[41]
**Activated charcoal**	M. blue	0.05-5	25.25	[42]
**Zeolite**	M. blue	-	10.82	[43]
**Rice husk**	M. blue	10-125	40.58	[44]
**Caulerpa cemosa Var.cylindracea**	M. blue	50	5.23	[44]
